# Evaluation of the Advanta Dx SARS-CoV-2 RT-PCR Assay, a High-Throughput Extraction-Free Diagnostic Test for the Detection of SARS-CoV-2 in Saliva: A Diagnostic Accuracy Study

**DOI:** 10.3390/diagnostics11101766

**Published:** 2021-09-26

**Authors:** Sofia Balaska, Dimitrios Pilalas, Anna Takardaki, Paraskevoula Koutra, Eleftheria Parasidou, Ioanna Gkeka, Areti Tychala, Georgios Meletis, Barbara Fyntanidou, Simeon Metallidis, Efthymia Protonotariou, Lemonia Skoura

**Affiliations:** 1 Department of Microbiology, AHEPA University Hospital, Medical School, Aristotle University οf Thessaloniki, 54636 Thessaloniki, Greece; smpalask@hotmail.com (S.B.); annatakardaki@gmail.com (A.T.); eviekou88@gmail.com (P.K.); pa-eleftheria@hotmail.com (E.P.); gkekagi@gmail.com (I.G.); aretich@gmail.com (A.T.); meletisg@hotmail.com (G.M.); protonotariou@gmail.com (E.P.); 2 First Propedeutic Department of Internal Medicine, AHEPA University Hospital, Medical School, Aristotle University of Thessaloniki, 54636 Thessaloniki, Greece; dpilalas@auth.gr; 3 Emergency Department, AHEPA University Hospital, 54636 Thessaloniki, Greece; bfyntan@yahoo.com; 4 First Department of Internal Medicine, Infectious Diseases Division, AHEPA University Hospital, Medical School, Aristotle University of Thessaloniki, 54636 Thessaloniki, Greece; metallidissimeon@yahoo.gr

**Keywords:** COVID-19, SARS-CoV-2, saliva, PCR

## Abstract

Nasopharyngeal swab specimen (NPS) molecular testing is considered the gold standard for SARS-CoV-2 detection. However, saliva is an attractive, noninvasive specimen alternative. The aim of the study was to evaluate the diagnostic accuracy of Advanta Dx SARS-CoV-2 RT-PCR saliva-based assay against paired NPS tested with either NeumoDx^TM^ SARS-CoV-2 assay or Abbott Real Time SARS-CoV-2 assay as the reference method. We prospectively evaluated the method in two settings: a diagnostic outpatient and a healthcare worker screening convenience sample, collected in November–December 2020. SARS-CoV-2 was detected in 27.7% (61/220) of diagnostic samples and in 5% (10/200) of screening samples. Overall, saliva test in diagnostic samples had a sensitivity of 88.5% (77.8–95.3%) and specificity of 98.1% (94.6–99.6%); in screening samples, the sensitivity was 90% (55.5–99.7%) and specificity 100% (98.1–100%). Our data suggests that the Fluidigm Advanta Dx RT-PCR saliva-based assay may be a reliable diagnostic tool for COVID-19 diagnosis in symptomatic individuals and screening asymptomatic healthcare workers.

## 1. Introduction

Large-scale testing has been one of the pillars in the effort to contain the spread of SARS-CoV-2 and quantitative reverse transcription PCR (RT-qPCR) has been established as the reference method [1]. RT-qPCR is performed on RNA extracted from upper respiratory specimens, such as nasopharyngeal (NPS), nasal mid-turbinate, or anterior nasal [2,3]. Saliva represents an appealing alternative as straightforward, non-invasive, painless procedure, which can be performed by the patient, requires minimal resources and does not necessitate healthcare worker supervision [4]. Current evidence supports the use of saliva for testing symptomatic individuals and repeated testing of asymptomatic individuals with a sensitivity comparable to nasopharyngeal specimens, which remain the gold standard [4].

The aim of this study was to evaluate the diagnostic accuracy of Advanta Dx SARS-CoV-2 RT-qPCR Assay, a saliva-based, extraction free testing method, against commercially available RT-PCR assays that use NPS for detection of the virus. We evaluated the assay diagnostic accuracy in two settings: diagnosing symptomatic patients and screening asymptomatic healthcare workers.

## 2. Materials and Methods

### 2.1. Study Design 

Prospectively collected paired NPS and saliva specimens submitted for SARS-CoV-2 molecular testing in November and December 2020 to the microbiology laboratory of AHEPA University Hospital, Thessaloniki, one of the reference hospitals for COVID-19 infection in Northern Greece, were included in the study. We analyzed convenience samples from two distinct adult patient populations: samples obtained from symptomatic outpatients (regional health centers and hospital dedicated clinic) presenting for diagnostic testing and samples from hospital healthcare workers (HCW) in the context of screening testing. Each patient contributed only once to the study. Assessors were not blinded to the results of the reference or the index test. Our report conforms to the Standards for Reporting Diagnostic Accuracy [5]. 

### 2.2. Data Collection

A minimum set of deidentified information was assembled for each patient including age, gender and patient group. Detailed clinical information collection was not consistent due to workload constraints and not further considered for study purposes.

### 2.3. Specimen Collection

The study participants were instructed to avoid the consumption of food and drinks, smoking, use of nasal sprays and practice of oral hygiene 30 min before sampling. A self- collected saliva sample of at least 2 mL was placed in a sterile falcon type tube or in a sterile urine collection container without HCW supervision. Next, an NPS was collected by trained HCWs. Swabs were soaked into transport media in the collection tube. Saliva specimens were stored in laboratory at room temperature and processed within 2 days of collection, while NPS were stored at 2–8 °C and processed within 24 h of collection. 

### 2.4. Specimen Analysis

All specimens were processed in the dedicated molecular diagnostics laboratory of the AHEPA University Hospital in Thessaloniki, Greece.

#### 2.4.1. Advanta Dx SARS-CoV-2 RT-PCR Assay (Fluidigm)

The Advanta™ Dx SARS-CoV-2 RT-PCR Assay (Fluidigm Corporation, South San Francisco, CA, USA), a saliva-based test, was established using Biomark HD device, a nanofluidic, automated, real-time PCR system that exploits the microfluidic technology through the use of dynamic arrays of integrated fluidic circuits (IFCs), enabling lower sample volume requirements and a high testing capacity. The assay has been authorized for use under FDA Emergency Use Authorization (EUA) and is CE-IVD marked under the In Vitro Diagnostics Directive (IVDD 98/79/EC). In brief, the workflow consists of the following steps: i. specimen preparation (dilution of saliva specimens in PBS and RNA secure), ii. heat inactivation, and iii. three consecutive reaction steps (RT, pre-amplification and qPCR). For both the 1-step RT-preamplification and real-time PCR detection steps, 2019-nCoV Real-Time RT-PCR Diagnostic Panel N1, N2, and RNase P (RP) primers and probes, developed by the United States Centers for Disease Control and Prevention (CDC), are employed. Moreover, three quality controls (“no template” control, negative extraction control and positive control) must run along with 93 samples on each 96-well sample-processing plate. Each control is treated in the same manner as the sample. 

The collected performance data were further analyzed using the Fluidigm Real-Time PCR Analysis software and Advanta Dx SARS-CoV-2 RT-PCR Assay Interpretive software (both from Fluidigm Corporation, South San Francisco, CA, USA) based on a Cycle threshold (Ct) 32 cut off.

With regard to analytical sensitivity, the Limit of Detection (LoD) for the Advanta Dx SARS-CoV-2 RT-PCR Assay was estimated to be 6.25 genome equivalents (GE)/μL according to the instructions for use document [6].

#### 2.4.2. Reference Standard

NeumoDx^TM^ SARS-CoV-2 Assay: NeumoDx^TM^ 96 system is a fully automated platform which performs extraction and amplification by Real-Time PCR in one device. Amplification targets are two conserved regions of the non-structural proteins (Nsp2) and N genes, PCR cut-off is set at 40 cycles and Limit of Detection (LoD) is reported to be at 150 copies/mL [7]. 

Abbott Real Time SARS-CoV-2 assay: Abbott Real Time SARS-CoV-2 assay targets two conserved regions of the RNA-dependent RNA polymerase (RdRp) and N genes with a reported LoD of 100 copies of viral RNA per milliliter of transport media, equate to 3.1 genome equivalent per reaction. Results are reported as positive if the Ct value is ≤31.5, based on the signal threshold determined by the manufacturer [8]. 

### 2.5. Sample Size Calculation

A sample size of 218 would be required to detect a 85% sensitivity with a 15% width 95% CI, assuming a 10% disease prevalence in the diagnostic and screening groups [9]. Of note, the study period coincided with the peak of the second wave of the COVID-19 pandemic, which disproportionately affected Northern Greece [10].

### 2.6. Data Analysis 

Categorical variables were expressed as proportions and continuous variables were expressed as median and interquartile range (IQR). NPS results were considered the reference standard. In case of an invalid PCR result with any method, the pair of samples was excluded.

Sensitivity, specificity, positive predictive value and negative predictive value were calculated with 95% exact confidence intervals (CI). Considering method failure as a distinct result category, we calculated the agreement between saliva and the NPS with Cohen’s kappa coefficient. Analyses were performed with R version 4.0.0.3 (R Foundation for Statistical Computing, Vienna, Austria—statistical packages binom, psych) [11,12,13].

## 3. Results

**Figure 1:**
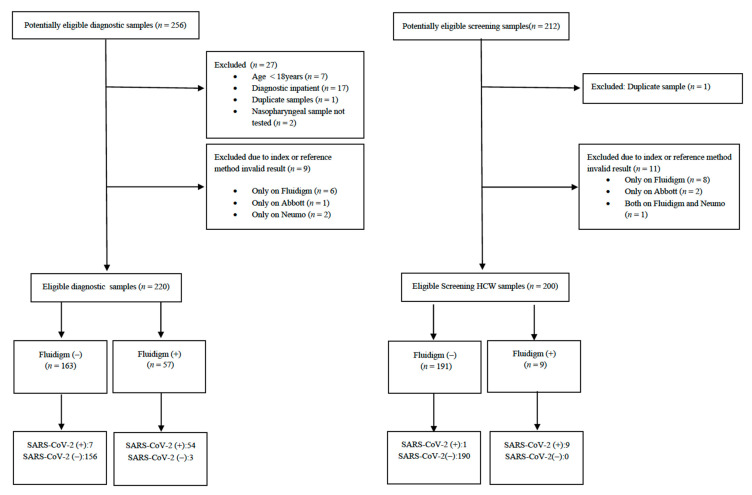
Diagnostic accuracy of Advanta™ Dx SARS-CoV-2 RT-PCR saliva-based assay versus paired nasopharyngeal specimen samples: study flowchart.

Paired NPS and saliva samples were obtained from patients during the study period; the study flowchart is presented in Figure 1. A total of 420 pairs of samples were further analyzed. Patient characteristics overall and by testing platform are presented in Table 1. COVID-19 prevalence, as estimated with the reference methods, was 27.7% (95% CI: 21.9–34.1%) in diagnostic samples and 5% (95% CI: 2.4–9%) in screening samples.

Overall, the saliva test sensitivity for diagnostic samples was 88.5% (95% CI: 77.8–95.3%) and 90% (95% CI: 55.5–99.7%) for screening samples. The method sensitivity, specificity, positive and negative predictive value by reference platform, and study population are presented in Table 2.

Including method failure as a category, the Cohen’s kappa was 0.80 (95% CI 0.72–0.88) in diagnostic samples and 0.63 (95% CI: 0.42–0.83) in screening samples.

## 4. Discussion

We conducted a prospective diagnostic accuracy study, which evaluated the Advanta™ Dx SARS-CoV-2 RT-PCR Assay on saliva against commercially available RT-qPCR systems used for the detection of SARS-CoV-2 in the context of diagnostic testing and screening. The method’s sensitivity and specificity was high in both settings: In a meta-analysis adjusting for NPS testing as an imperfect reference standard, the NPS and saliva specimen assay sensitivity (84.8%, 95% Credible Interval, CrI: 76.8–92.4% versus 83.2%, 95% CrI: 77.4–91.4%) and specificity (98.9%, 95% CrI: 97.4–99.8% versus 99.2%, 95% 98.2–99.8%) were comparable [14]. The sample size was inadequate to allow for comparisons stratified by reference NPS assay.

It should be noted that, in the aforementioned meta-analysis, the eligible studies concerned ambulatory patients (15/16) and approximately half (9/16) were from an outpatient population with mild or no symptoms [14]; thus, we believe the aforementioned results are an appropriate benchmark to evaluate our results in both the diagnostic and screening testing settings.

COVID-19 infection presents an ongoing public health threat, and the emergence and spread of SARS-CoV-2 variants may be accompanied by a resistance to the immunity elicited by the existing vaccines [15]. Widespread testing will remain one of the core strategies to monitor the burden of disease and guide public health measures. The use of self-collected saliva specimens is less cumbersome for the patient, given that testing is likely to occur repeatedly and requires minimal resources. Saliva samples are very stable at room temperature, easy to store and provide consistent results in repeated testing [16]. Furthermore, evidence suggests that saliva testing may be more cost-effective compared to the testing of nasopharyngeal samples [17] The Fluidigm saliva-based assay protocol combines a high sensitivity with a relatively short turnaround time, while being highly scalable. However, limitations of the assay include the need for dedicated equipment and further clinical studies to expand the evidence base.

The major limitations of our study include the lack of detailed clinical data and the use of an imperfect reference standard, which consisted of two different assays used in the clinical routine. Furthermore, the specimen collection method was uniform, and we did not evaluate the impact of different specimen collection parameters on the diagnostic accuracy of the assay. No in vitro evaluation of the assay was performed, as this was beyond the scope of our study. Of note, all the samples were collected before the spread of the B.1.1.7 SARS-CoV-2 variant, and subsequent major variants of concern in Northern Greece, based on genomic analysis deployed by the National Genomic Surveillance Network (unpublished data). The emergence of new SARS-CoV-2 variants dictates constant vigilance with regard to the accuracy of our diagnostic tools; in silico analysis may provide invaluable insights in this setting [18].

## 5. Conclusions

We evaluated, for the first time, the diagnostic accuracy of the Fluidigm Advanta Dx RT-PCR saliva-based assay. Our study suggests that it may be a reliable solution to SARS-CoV-2 testing both from a single patient and a massive testing standpoint and confirm saliva as a non-inferior specimen for SARS-CoV-2 testing compared to NPS. Larger-scale studies are urgently needed to evaluate the method’s performance and applicability in more diverse scenarios and in the context of the spread of SARS-CoV-2 variants.

## Figures and Tables

**Table 1:** Demographic characteristics of the study population. SD: standard deviation.

	Overall	NeuMoDx	Abbott
Sample size	420	133	287
Mean Age (SD), years	44.7 (13)	43.6 (12.6)	45.2 (13.2)
Gender, *n* (%)			
Male	161 (38.3%)	49 (36.8%)	112 (39%)
Female	259 (61.7%)	84 (63.2%)	175 (61%)
Sample type, *n* (%)			
Diagnostic	220 (52.4%)	58 (43.6%)	162 (56.4%)
Screening	200 (47.6%)	75 (56.4%)	125 (43.6%)

**Table 2:** Diagnostic performance of Fluidigm across patient groups.

Patient Group	TP	FP	TN	FN	Sensitivity (95% CI)	Specificity (95% CI)	PPV (95% CI)	NPV (95% CI)
Diagnostic samples	54	3	156	7	88.5% (77.8–95.3%)	98.1% (94.6–99.6%)	94.7% (85.4–98.9%)	95.7% (91.4–98.3%)
Tested on NeuMoDx	17	0	40	1	94.4% (72.7–99.9%)	100%% (91.2–100%)	100% (80.5–100%)	97.6% (87.1–99.9%)
Tested on Abbott	37	3	116	6	86% (72.1–94.7%)	97.5% (92.8–99.5%)	92.5% (79.6–98.4%)	95.1% (89.6–98.2%)
Screening samples	9	0	190	1	90% (55.5–99.7%)	100% (98.1–100%)	100% (66.4–100%)	99.5% (97.1–100%)
Tested on NeuMo	3	0	72	0	100% (29.2–100%)	100% (95–100%)	100% (29.2–100%)	100% (95–100%)
Tested on Abbott	6	0	118	1	85.7% (42.1–99.6%)	100% (96.9–100%)	100% (54.1–100%)	99.2% (95.4–100%)

TP: true positive, FP: false positive, TN: true negative, FN: false negative, CI: confidence interval, PPV: positive predictive value, NPV: negative predictive value, HCW: healthcare workers.

## Data Availability

Data allowing reproducing the diagnostic accuracy evaluation are included in the Table 2 of the Article.
